# Beyond MHz image recordings using LEDs and the FRAME concept

**DOI:** 10.1038/s41598-020-73738-1

**Published:** 2020-10-06

**Authors:** Vassily Kornienko, Elias Kristensson, Andreas Ehn, Antoine Fourriere, Edouard Berrocal

**Affiliations:** 1 Department of Combustion Physics, Lund University, Lund, Sweden; 2 Federal Institute for Geosciences and Natural Resources (BGR), Hanover, Germany

**Keywords:** Imaging and sensing, Imaging techniques

## Abstract

Many important scientific questions in physics, chemistry and biology rely on high-speed optical imaging techniques for their investigations. These techniques are either passive, relying on the rapid readout of photoactive elements, or active, relying on the illumination properties of specially designed pulse trains. Currently, MHz imaging speeds are difficult to realize; passive methods, being dictated by electronics, cause the unification of high spatial resolution with high frame rates to be very challenging, while active methods rely on expensive and complex hardware such as femto- and picosecond laser sources. Here we present an accessible temporally resolved imaging system for shadowgraphy based on multiplexed LED illumination that is capable of producing four images at MHz frame rates. Furthermore as the LEDs are independent of each other, any light burst configuration can be obtained, allowing for instance the simultaneous determination of low- and high speed events in parallel. To the best of the authors’ knowledge, this is the fastest high speed imaging system that does not rely on pulsed lasers or fast detectors, in this case reaching up to 4.56 MHz.

## Introduction

High-speed imaging and videography is important for a plethora of measurement situations such as for investigating chemical reactions^1^, fuel injection^2,3^, combustion^4,5^, plasma formation^6,7^ and laser induced material damage^8–10^. The first instance of kHz imaging (2 kHz) can actually be traced back to 1927 when Edward G. Beardsley illuminated a fuel spray from an oil engine injection with 20 individual sparks in the Langley Research Center of the National Advisory Committee for Aeronautics (NACA, the precursor of NASA)^11^. This being top secret work, 10 years later, Edgerton and Germeshausen were able to achieve a similar feat at a speed of 6 kHz, applying their work to filming both sprays and aircraft propellers^12^. During the early 1980s, before the dawn of digital storage, high-speed video cameras used either analog recording in magnetic tape (Kodak Spin Physics SP2000) or rotating mirrors imaging the event on photographic film. In 1991 Takeharu Etoh and Kodak invented the Ektapro line of cameras, of which the first one was capable of imaging at a speed of $40\times 10^3$ frames per second (fps) for $64 \times 64$ pixels (a partial readout scheme on $256 \times 256$ pixels)^13^. This has opened the doors for the development of fast digital passive imaging technology, i.e. imaging where light emitted from an event is recorded on a 2D array of photoactive elements^14^. Applications of this camera include high-speed imaging of diesel fuel sprays^15^ and obtaining particle image velocimetry (PIV) maps of the flow within an engine cylinder^16^.

The next major improvement within passive high-speed imaging came with the in situ storage image sensors (ISIS), where the images are stored on the CCD chip before reading out. The temporal resolution of such sensors is thus limited by the electron transfer speed between CCD elements instead of the bottleneck readout speed. However, this also means that the video length cannot be longer than the number of storage elements that are reserved for a given pixel. The first burst image sensor of this kind had a frame count of 30 frames, a pixel count of $360 \times 360$ and a speed of 1 Mfps^17^. The frame rate culmination of ISIS technology came in 2005, upon the creation of a CCD with a readout rate of 100 Mfps and a sequence depth of 16 frames^18^. However, to achieve this performance the image size was reduced to $64 \times 64$ pixels. Currently available state-of-the-art high-speed cameras can provide ~ 1 Mpixel pixel resolution at Mfps speeds^19,20^ and their applicability is essentially only limited by their cost.

In general for passive high-speed digital imaging, the necessity of in situ storage makes it difficult to attain high pixel numbers. This necessity leads to a trade-off between frame rate, spatial resolution and number of images (sequence depth). Silicon sensors also have an intrinsic theoretical speed limit of 90 Gfps caused by the mixing of electrons originating from the varying penetration depths of different wavelengths^21^. However, this theoretical limit has thus far not been achieved, partially due to another challenge associated with high-speed imaging: as imaging speeds increase, passive imaging techniques fall victim to an insufficient amount of light incident on the sensors during the short exposure time. An increased sensor sensitivity could compensate for the reduction in the signal level yet with currently available technology this would imply increasing the photoactive area, yielding lower frame rates. A possible way to circumvent these problems and still attain high imaging speeds is to apply computational imaging approaches to regular cameras. Using an off-the-shelf cMOS camera in conjunction with a DMD and galvoscanner, the COSUP technique has demonstrated just this, with a 1.5 Mfps video comprising of 500, 0.5 Mpixel frames^22^. However, due to mechanical constraints on the galvo, higher speeds have not been demonstrated without incorporating another level of complexity in the form of a streak camera^23^. Thus, the development of faster imaging systems has steered into laser illumination-based (*active*) imaging concepts^24–28^. In contrast to passive high-speed imaging, active techniques illuminate 2D transient events with specially designed pulse trains in order to capture the dynamics. This shift to the illumination side allows for a boost in signal level and the ability to surpass the detector speed limit. However, all current demonstrations of these ultrafast methods require either pico- or femtosecond laser systems and thus cannot be scaled down to the MHz level without significantly altering their optical design. Hence the technology currently available for high speed imaging in the MHz regime mainly relies on systems—passive or active—that use advanced and/or expensive hardware.

In this paper we present an accessible LED-based fast illumination system, which, in combination with off-the-shelf camera technology, can image at a speed of 4.56 Mfps (limited by the pulse duration of current LED technology) while still maintaining both high spatial resolution and sensitivity.

## Method

The illumination system presented here is a back-lit (transillumination) configuration based on the FRAME (Frequency Recognition Algorithm for Multiple Exposures) technique^27^. A time-resolved back-lit imaging system captures a snapshot of the light-obstruction (shadow) caused by the probed object, which casts a silhouette image upon a camera sensor. The presented FRAME-based configuration allows for a rapid succession of four such images to be acquired within a single camera exposure. By applying unique spatial modulations to the individual pulses of the pulse train, the shadow of the object at each instance in time becomes tagged during the light-matter interaction. These timestamped shadow images are then extracted using a spatial lock-in algorithm on the acquired data.

### Frequency recognition algorithm for multiple exposures

**Figure 1:**
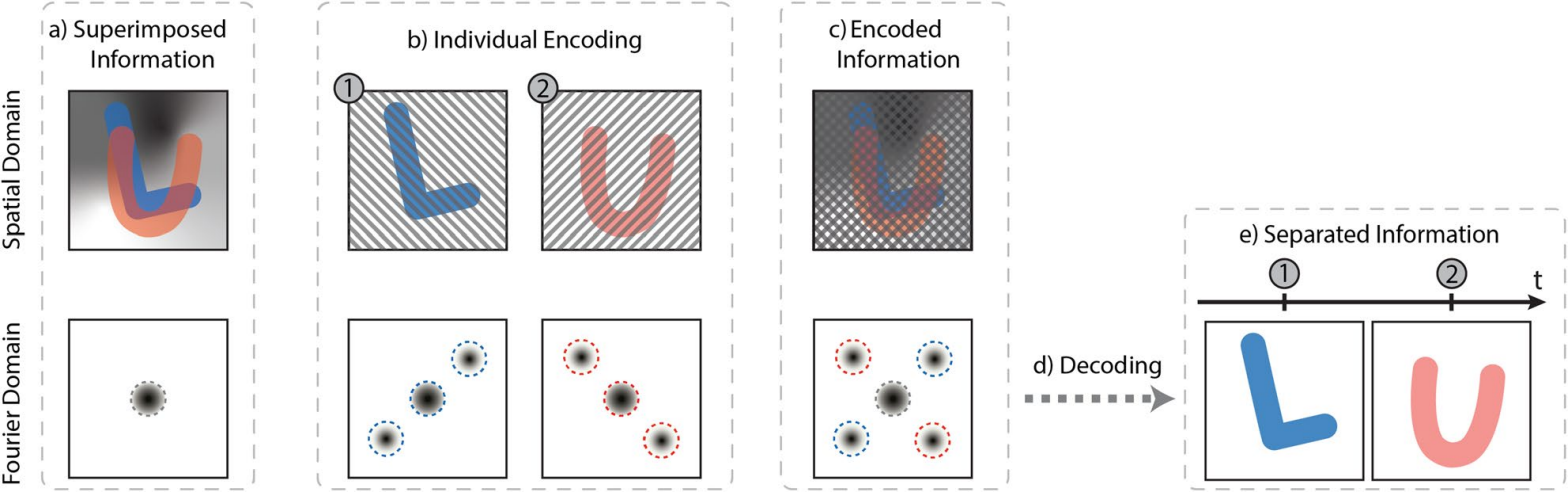
The working principle of FRAME. With no applied spatial modulation, all the image information (the L and U along with an independent background light field) is superimposed and centered in the Fourier domain (**a**). Upon application of unique spatial modulations, the individual image information is shifted into empty areas of the Fourier domain (**b**), allowing for the separation of the timestamped snapshots taken within one exposure (**c**). Applying a spatial lock-in algorithm followed by a low pass filter (**d**), the individual images can then be extracted, with a suppressed background light field (**e**).

Natural images are characteristically sparse in their spatial frequency (Fourier) domain, only utilizing a small fraction of the reciprocal space available to high resolution cameras (Fig. 1a). By applying a spatial modulation to an image, its information can be shifted into these otherwise empty areas of the Fourier domain (Fig. 1b), forming what is referred to as a Fourier cluster. When a set of uniquely spatially modulated images are recorded on the same sensor, the Fourier transform of the recorded image will thus exhibit several Fourier clusters (Fig. 1c). Since the spread of each cluster is small, the respective image information they carry does not overlap. A single photograph can thus contain a full set of images—a multiplexed image series. Using a lock-in algorithm followed by a low-pass filter^27,29,30^ (Fig. 1d), the individual clusters can be isolated and the image information contained therein extracted (Fig. 1e). Thus, by implementing this spatial coding strategy, one can store multiple temporally separated observations of an event in a single exposure and extract them into an image sequence.

When FRAME is implemented for high-speed imaging, the coded information corresponds to a pulse train of uniquely spatially modulated light pulses probing an event. The event is then recorded in its entirety on a camera within a single exposure, i.e. all the coded image information is superimposed on the sensor. Since each pulse interacted with the sample at different times (the “L” at time 1 and the “U” at time 2 w.r.t. Fig. 1), their respective Fourier clusters will correspond to a timestamped snapshot of the event. The attainable temporal resolution of the imaging system has thus been shifted from the electronics of the camera to the illumination characteristics of the pulse train. As a result, high speed imaging without the constraint on camera pixel number or sensitivity is made possible. In conjunction with a femtosecond laser source, this technique has demonstrated a 5 Tfps image sequence of a light pulse traveling through a Kerr medium^27^. Although Tfps speeds can be attained, ultrafast laser technology is not a fundamental necessity for FRAME to function. Indeed, the application of spatial modulations, paramount to the functioning of FRAME, is independent of light source. This choice, be it e.g. narrowband lasers or broadband LEDs, can be completely determined by the type of event to be probed. Furthermore the tagging is not solely limited to the temporal domain but also for the tagging of any image dimension, opening up for snapshot multispectral-^29^, polarization-^29^ and 3D-imaging^29,30^.

This method of tagging images also carries with it another useful property, namely background light suppression. If the background grayscale light field of Fig. 1a is independent of the image information (independent of the L and U, as in Fig. 1b), it will remain unmodulated. The low-pass filter will hence effectively eliminate it, allowing for a higher signal to background ratio in the final extracted images (Fig. 1e and Supplementary Information 6).

### Experimental setup

The aim of the presented work is to demonstrate the possibility of achieving high-resolution MHz imaging, without relying on advanced and expensive hardware such as time-gated detectors, streak cameras and/or pulsed lasers. Instead, the illumination system presented herein (a prototype of the FRAME Illumination Unit developed in collaboration with LaVision GmbH) consists of four Ce:YAG white light LEDs (Cree XHP35) that can be switched on and off on a sub-microsecond time scale (rise time of 205 ns, from 10 to 90% of full power, and temporal jitter FWHM of 34.5 ns—see Supplementary Information 7). Each LED is controlled individually, which not only allows for adjustable frame rates from $10^{0}$ to $10^{7}$ fps but also for more complex non-linear triggering sequences and variable brightness. Furthermore, by implementing the FRAME image-coding methodology, there is no need for fast image readout, which allows us to capture the transillumination frames using slow, yet sensitive high-resolution detectors. This is demonstrated here using a standard 10-bit CMOS 5 MP camera (LaVision, Imager M-lite) mounted with a telecentric objective lens (Edmund Optics Gold TL 1X or 0.5X).

**Figure 2:**
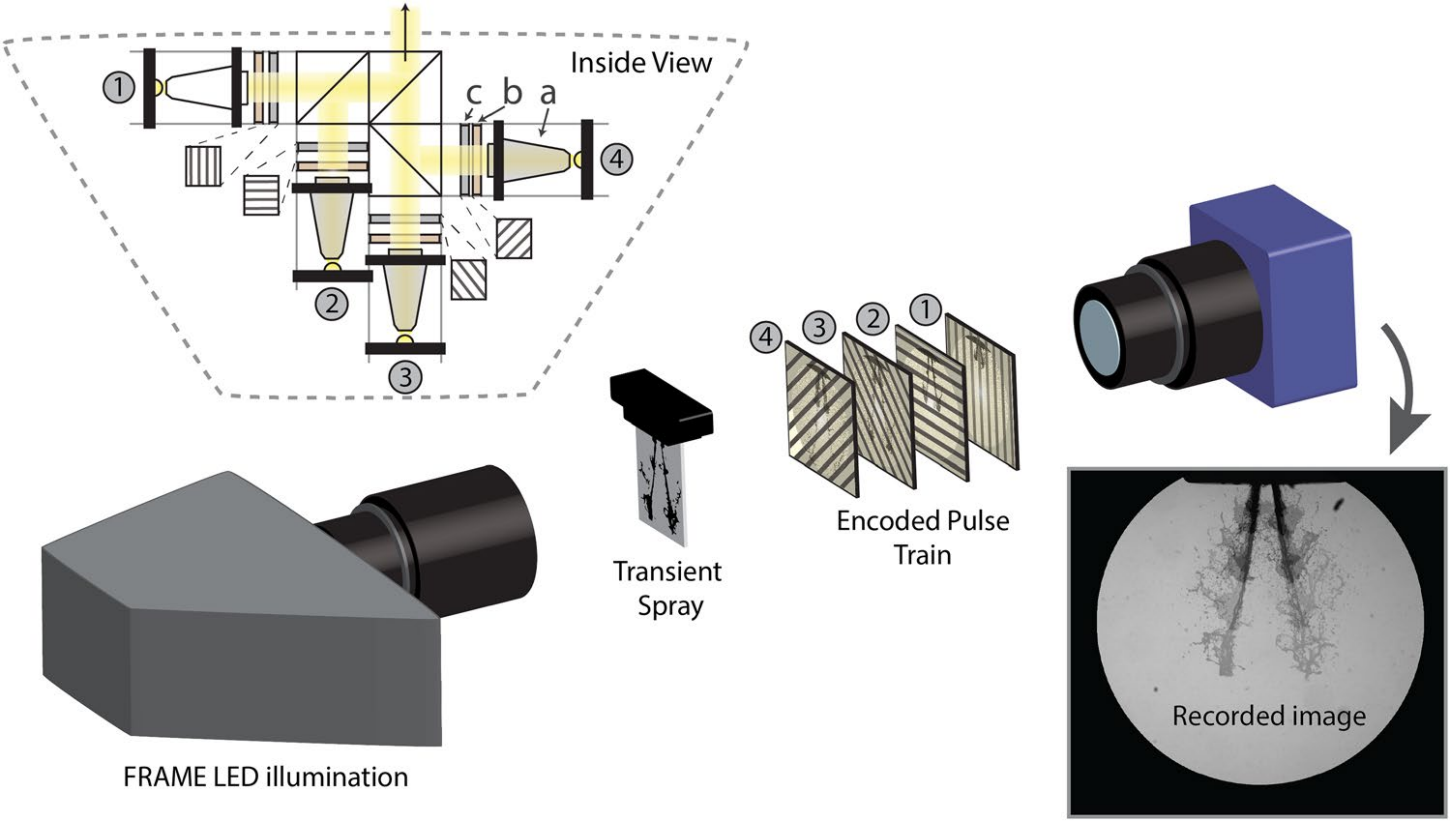
Recording an injection event with FRAME. The light from four pulsed white-light LEDs are led through individual optical arms consisting of a tapered light pipe with 3$\times$ magnification (**a**), a diffusor (**b**) and a Ronchi grating (**c**). Beamsplitters recombine these into a collinear pulse train. After passing through an objective lens, they illuminate the sample with a spatially modulated pulse train (indicated with numbers). The pulse train is subsequently recorded on a camera of choice within a single exposure.

The optical configuration of the system is schematically illustrated in Fig. 2. The light from each LED is guided along an optical arm consisting of a tapered light pipe with 3× magnification (a), a diffusor (b), and a Ronchi grating (50 lp/mm) rotated to a unique angle (c). The four spatially encoded white light pulses are thereafter combined into a collinear pulse train using beam splitters. A Nikon 50–300 mm camera objective is used to image the gratings onto the sample, permitting the magnification of the illuminated area—the viewed area—to be readily modified. In our study a commercially available fuel port injector, Bosch EV1 4-holes nozzle^31^, with orifice size of 280 μm, running at 4.8 bar water injection pressure, was probed at different times after start of injection (ASOI). Its complex transient structures allows us to demonstrate the ability of our system in obtaining images with both high spatial- and temporal resolution.

**Figure 3:**
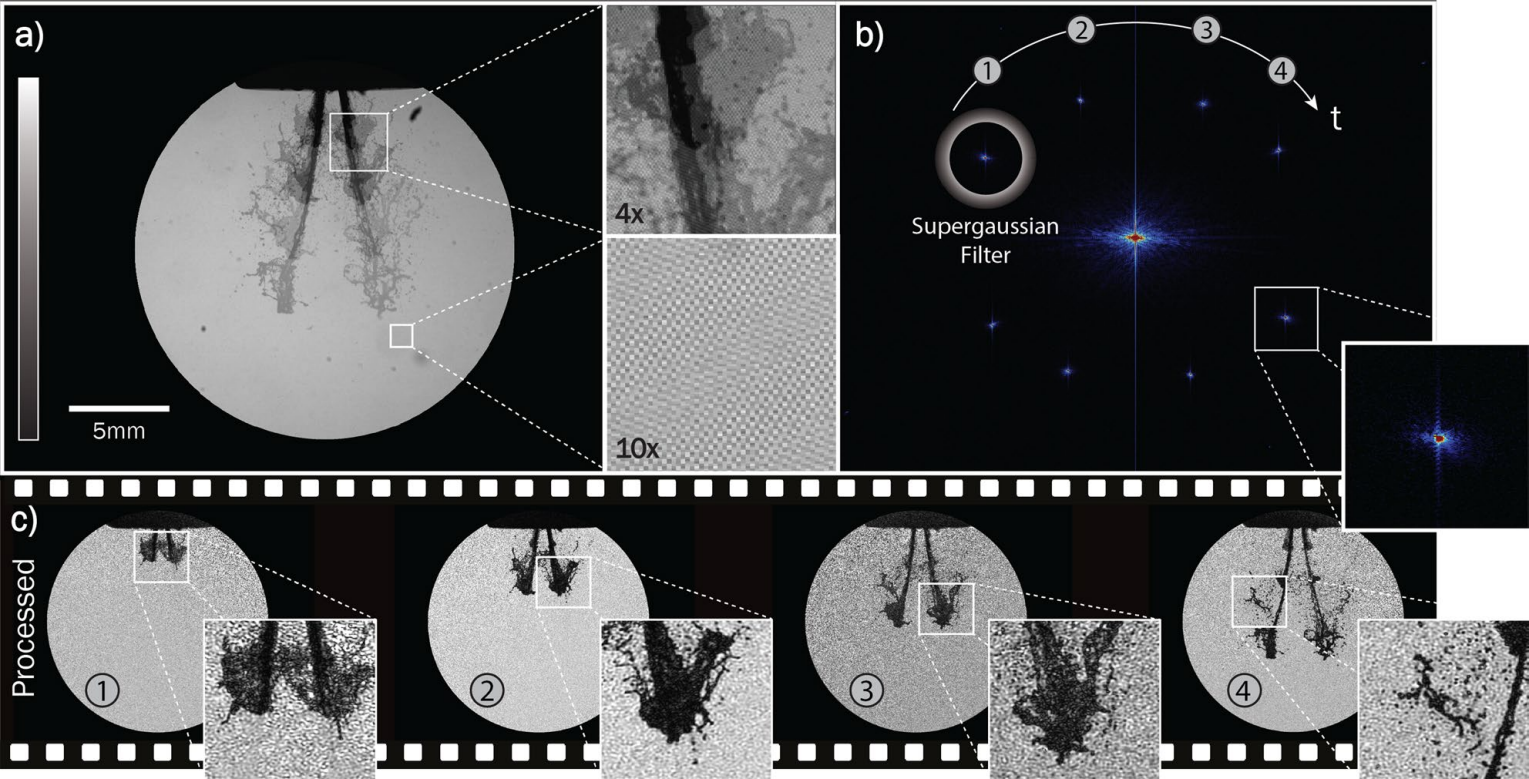
The extraction process of a 20 kHz image series. (**a**) The single exposure (raw) image with the four uniquely modulated pulses summed onto the camera sensor. The crosshatch pattern of the superimposed modulations is highlighted in the 10$\times$ zoom. (**b**) The Fourier transform of (**a**) where each Fourier cluster holds the image information from a single pulse. (**c**) The extracted, background-corrected image series, where the zoom-ins highlight the visibility of fine structures.

During an acquisition, the spray is illuminated with the four intensity-modulated pulses and the transmitted light from all pulses is summed on the camera sensor within a single exposure (Fig. 3a). The magnifications in Fig. 3a show the resulting crosshatch pattern of the overlapping modulated structures (10$\times$ zoom) and the various grayscale shadows of the spray at different times as the pulse train illuminates it (4$\times$ zoom). It can be seen that the dynamic range of the sensor has been divided among the four illuminating pulses, resulting in gradually lighter shadows in the pixel positions the spray covers as it evolves. Here, in the spatial domain, the image information for the individual events overlap and cannot be accessed. However, the angle and frequency of the Ronchi gratings have been chosen such that their corresponding Fourier clusters are placed as far away from adjacent clusters and recurrent/static structures in reciprocal space as possible (Fig. 3b). The lock-in algorithm subsequently shifts each cluster to the center of the Fourier domain, where the data is low-pass filtered (2D-Gaussian to the power of 8) and finally inverse Fourier transformed back to the spatial domain. The images are then background corrected, resulting in the final temporally resolved image series (Fig. 3c).

### Spatial resolution

The spatial resolution of an imaging system can be deduced by measuring its modulation transfer function (MTF). In practice, this involves imaging a Sector Star target (Siemens target), from which the system’s cut-off spatial frequency is extracted, i.e. the number of line-pairs per millimeter (lp/mm) the system can *spatially resolve* in an arbitrary direction. According to the Rayleigh criteria, a MTF contrast of 15.3% is required to resolve two neighboring points sources and is often the value used to define the spatial resolution of an imaging system. Specifying the system’s spatial resolution alone is, however, not enough to assess its imaging capabilities; it is equally meaningful to specify the viewing area or field-of-view (FOV) over which the measured spatial resolution is maintained. Here we define two parameters that take into account the relevant information concerning spatial resolution: (1) the maximum number of resolvable line-pairs per unit of length (*RLP*) and (2) the total number of resolvable line-pairs (*NLP*) across the FOV where the resolution, *RLP*, is maintained. In the text we will combine the two parameters, *RLP* and *NLP*, in a condensed form specified accordingly: (*RLP*:*NLP*)$_{X}$, where *X* indicates that the numbers refer to the *X* dimension. Using this nomenclature, an image with a spatial resolution of 60 lp/mm and a FOV of 1 cm in the y-direction would thus be written as (60 lp/mm:600)$_Y$.

### Temporal resolution

In contrast to most passive high speed imaging techniques, image series acquired using illumination-based systems can have frames that overlap in time, i.e. an acquisition rate of frames exceeding the inverse of the frame duration (integration time). The parameter governing the system’s temporal resolution—interframe time or frame duration—may therefore vary depending on the type of system^32^ and, hence, it becomes useful to clarify the term “temporally resolved” for high-speed imaging, in particular for illumination-based systems.

**Figure 4:**
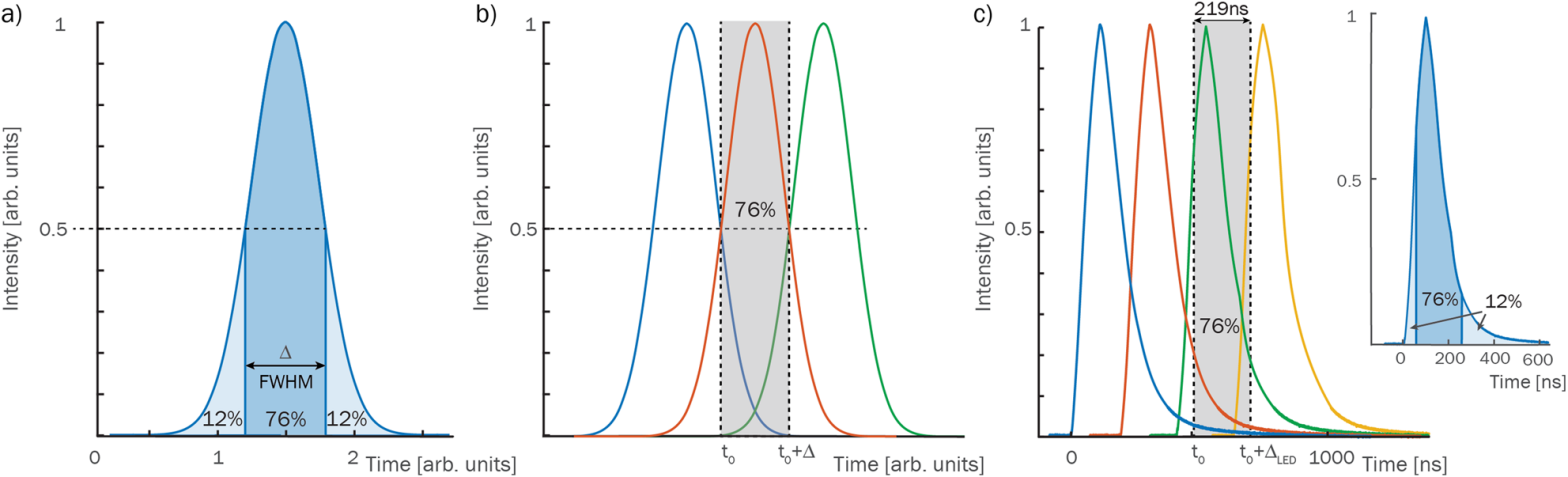
Temporal resolution of an imaging system. (**a**) A Gaussian gate profile has $76\%$ of its power concentrated within its FWHM. Placing individual Gaussian pulses in a pulse train such that their half maxima limits coincide, (**b**), assures that 76% of the information generated by, for example, the red pulse is temporally unique to the time interval $[t_0,t_{0} + \Delta ]$, where $\Delta = FWHM$. Using this $\Delta$ as the discretizing step size on the time-axis, we consider the resulting pulse train *temporally resolved*. (**c**) Generalization of this concept to the temporal gate profiles of the LED pulses produced by the system presented herein leads to temporal limits within which 76% of the pulse’s power resides. Discretizing the time-axis with these limits (corresponding to a step size of $\Delta _{\text {LED}} = 219$  ns) results in a maximum imaging speed, or maximum sampling rate, of 4.56 MHz.

The purpose of all video systems is to extract a sequence of images that shows the temporal evolution of an event. Most often this means sampling the time axis with image frames at discrete points, where each frame has a set integration time ($\Delta$). Even if illumination-based imaging systems have the ability to overlap image frames in time, according to the Nyqvist samling rate theorem^33^ this approach does not provide additional information about the event’s temporal evolution. To avoid such oversampling of data, the frames must instead be arranged so that they are temporally separated. In order to determine when two or more frames are considered temporally separated, we propose an analysis based on image frames with Gaussian temporal gate functions. 76% of the image information contained within such a frame can be traced to the time-slot bounded by its FWHM (the $\Delta$ of Fig. 4a). Thus, by arranging these such that their FWHMs coincide (Fig. 4b), the time axis becomes discretized such that each sampled time-slot is associated with a given image to a degree of 76%. This strategy of sampling the time-axis—discretizing it with a step size of $\Delta$—guarantees a 76% degree of “temporal uniqueness” to each image, it is generalizable to any temporal gate profile and avoids oversampling of the event. The inverse of the step size, $1/\Delta$, is then the maximum attainable sampling rate of the event’s temporal evolution, i.e. the maximum imaging speed of the system.

The temporal profiles of the frames acquired using illumination-based imaging systems can be determined from the illuminating pulse train. For the present system, we measure the shortest attainable such profile of the LED pulses using a photodiode and 6 GHz oscilloscope (inset of Fig. 4c)—this is what will set maximum the sampling rate of the system. Here, equivalently to the Gaussian profiles, an upper and lower limit has been extracted within which 76% of the pulse power is concentrated. This $\Delta _{\text {LED}}$ of 219 ns is used as the discretizer of the temporal evolution of the event, resulting in a maximum temporal imaging speed of 1/219 ns $= 4.56$ MHz associated with the system presented herein (see Supplementary Information 4 for more measurement examples).

As for the description of spatial properties, the description of the temporal properties of an acquired image series requires, at a minimum, (1) the sequence length (*SL*), specified in time and (2) the sequence depth (*SD*), i.e. number of *temporally resolved* images over the time *SL*. To specify these in a condensed form, the same notation as earlier will be used for temporal properties: $(SL:SD)_T$ (where *T* indicates that the numbers refer to the time dimension). Using this nomenclature, an image series consisting of 13 temporally resolved frames acquired within 1500 μs would thus be written (1500 μs:13)$_T$. For the system presented herein, the sequence depth is constant at four frames and the shortest presented sequence length is 876 ns, i.e. (876ns:4)$_T$, corresponding to 4.56 Mfps (Fig. 4c).

## Results

Unlike most high speed imaging systems, the current system can achieve frame rates in the MHz-regime without compromising FOV or sensitivity. Furthermore, since the illumination dictates the properties of the time axis, the image series can be tailored from the Hz to the MHz regime or a mixture of both. Variable magnification is also a possibility and dependent solely on the optics chosen. Here we present measurements that illustrate these capabilities.

### Temporally resolved imaging at kHz to MHz frame rates

**Figure 5:**
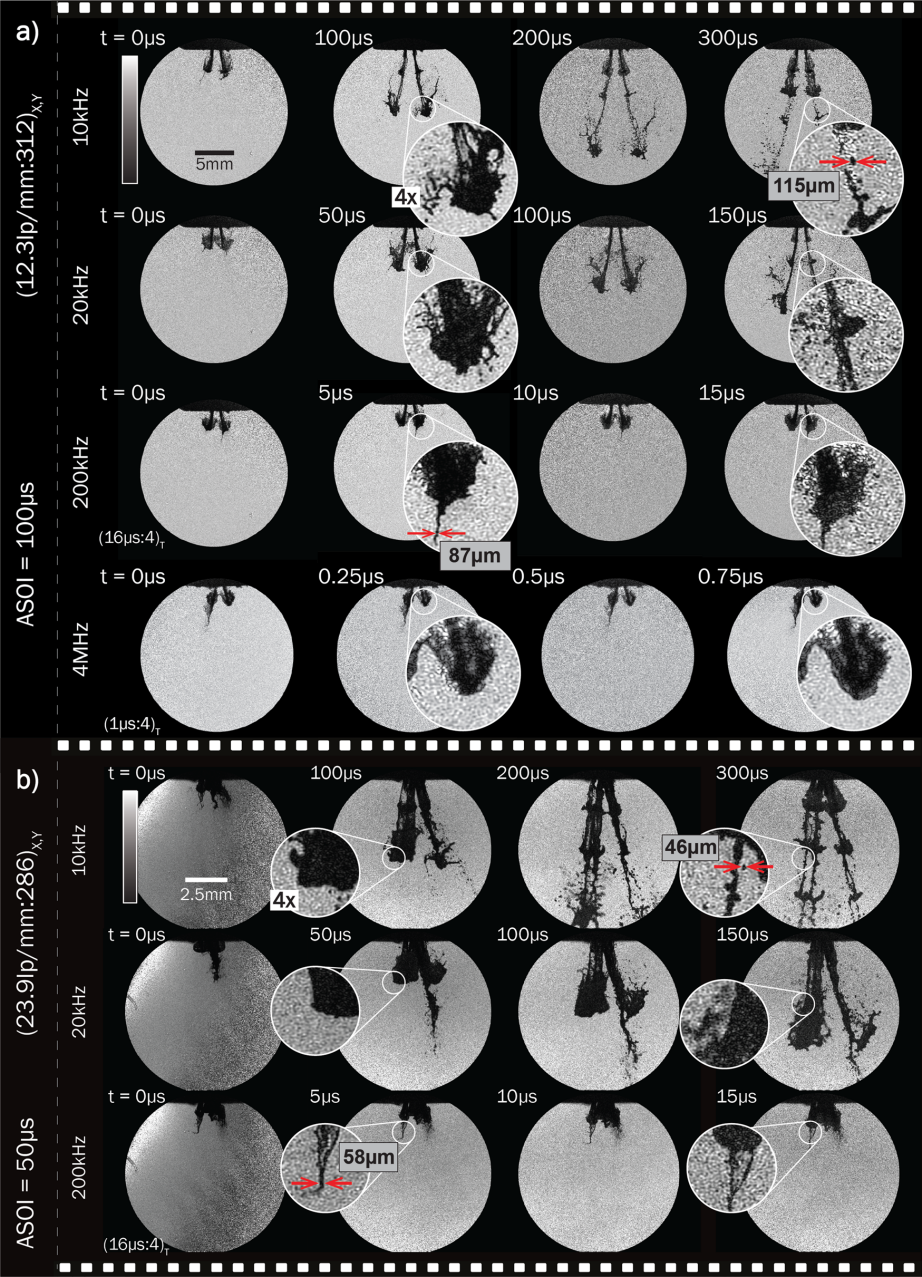
The versatility of speeds and magnifications allowed by the system. (**a**) shows a collection of series taken 100 μs after start of injection (ASOI) with a large FOV at speeds ranging from 10 kHz to 4 MHz. (**b**) is a collection of series taken at 50 μs ASOI and small FOV. The zoom-ins depict identical positions at different times, emphasizing the movement of the injection front at the given timestamps.

A set of single-exposure images was acquired at different speeds and FOVs using the FRAME illumination system. Upon extraction, these resulted in multiple image series of the event, with speeds ranging from 10 kHz to 4 MHz (Fig. 5).

Since the pulse duration for the LEDs is variable and each is controlled individually, the illumination can be optimized with regards to the sample under study. The atomization process studied herein required ~ 1 μs in order to be temporally frozen. Increasing the duration of the illumination boosts the signal level but generates motion blur (for the current sample), whereas imaging at frame rates above 1 Mfps requires a reduction of the illumination time.

For a FRAME sequence consisting of frames with no temporally overlapping information, the total dynamic range of the sensor will be divided equally among each unique time-slot. In the current case, this would yield $2^{10}/4 = 256$ temporally unique grayscale levels per image. However, according to the definition stated above, a sequence can still be temporally resolved even if the frames partially overlap in time—at the temporal resolution limit there will be a $24\%$ information overlap within each time-slot. In the case of the 4 MHz image series of Fig. 5, this fact results in a decrease to 194 temporally unique grayscale levels per image, while the complete lack of overlap between the frames in the 10kHz series, results in no decrease of dynamic range per image (see Supplementary Information 8).

The presented set of measurements shows the possibility of probing a given event at various frame rates, allowing for easy access to many different types of dynamic samples. Furthermore, the ease with which the frame rate is interchangeable, by simply changing LED trigger times, allows the temporal sampling of the time-axis to be tailored to fit the object under study.

### Preservation of the field of view

For the measurements presented herein, an average spatial resolution for the individual extracted frames was measured under the Rayleigh criteria (contrast of 15.3%), resulting in 12.3 lp/mm and 23.9 lp/mm for the large and small FOVs respectively, i.e, (12.3 lp/mm:312)$_{X,Y}$ and (23.9 lp/mm:286)$_{X,Y}$ (see Supplementary Information 2 for the measurements).

**Figure 6:**
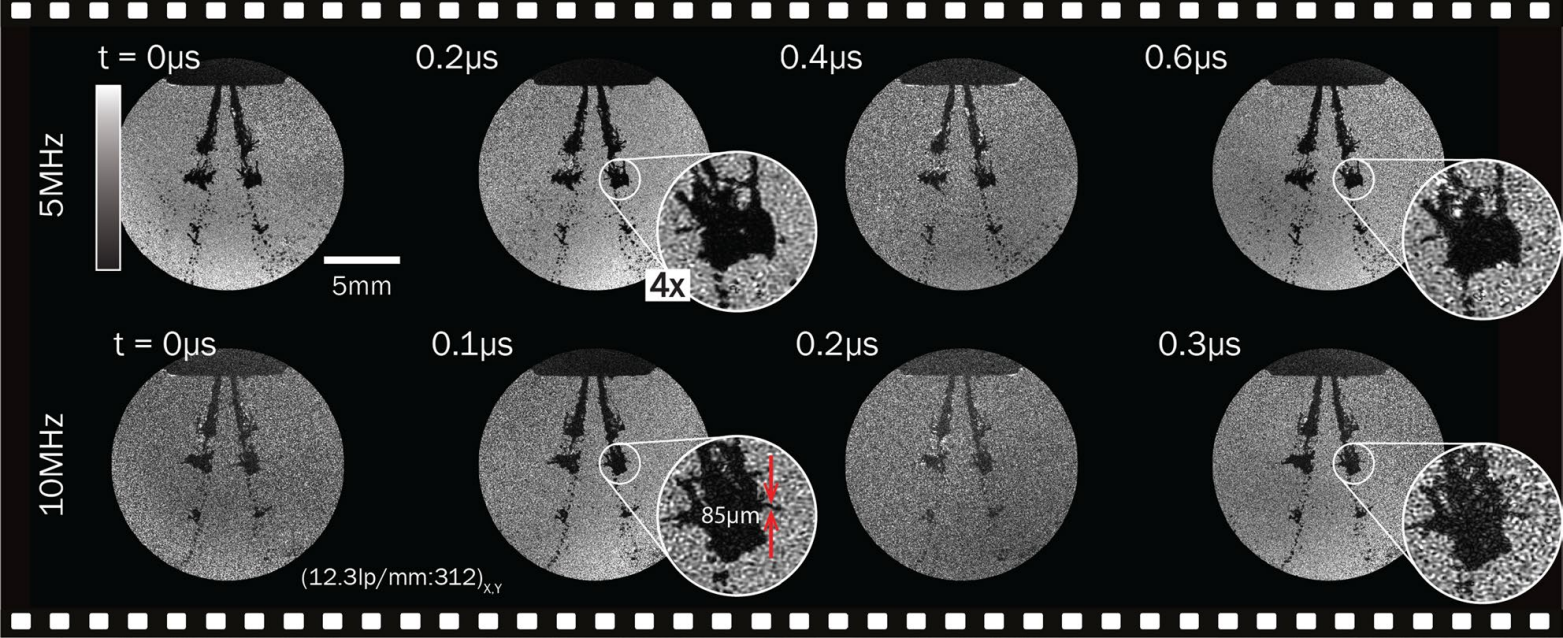
The preservation of FOV at high speeds. A set of image series of the injection event taken at speeds of 5 and 10 MHz, 2.3 ms ASOI. Fine structures are still equally visible for these higher speeds. Measurements on a Sector Star target show no degradation of the spatial resolution at these frame rates, i.e. (12.3 lp/mm:312)$_{X,Y}$ is preserved.

As has been mentioned earlier, the fixed pixel readout rate causes many passive techniques to suffer from a trade-off between speed and field of view. Since the videography speed of FRAME is completely determined by the illumination properties, the spatial resolution and FOV can be preserved independently of frame-rate. As a proof of concept two separate pulse trains consisting of four 200 ns pulses and four 100 ns pulses forming *non-temporally* resolved 5 and 10 MHz image series respectively, show the visibility of fine structures (Fig. 6). Indeed, measurements performed on a Sector Star target confirm that the system’s FOV and spatial resolution does not degrade with speed, even when exceeding the limit of the system’s maximum frame rate (see Supplementary Information 3 and 5).

### Various timestamp configurations

**Figure 7:**
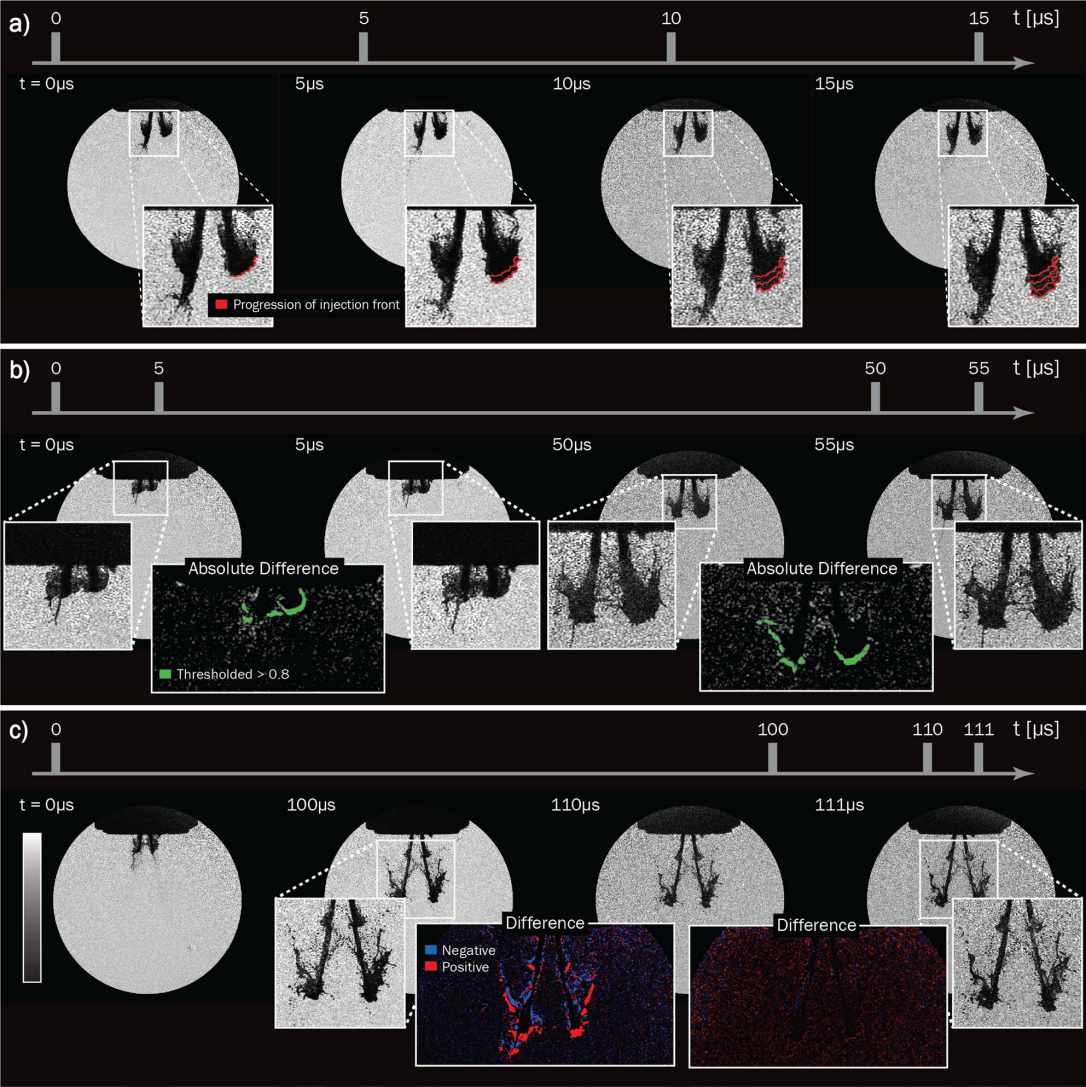
Illustration of the versatility in the time domain. (**a**) Shows a series depicting the leading edge of the injection (red) tracked at a constant inter-frame time. (**b**) Depicts a PIV-inspired event where two fast images were taken within 5 μs followed by a delay of 45 μs before another double trigger event was acquired. One can see that the green, representing the thresholded absolute difference between the images to its left and right, is approximately the same size for the two pairs of light pulses, corresponding to an injection front speed of about 40 m/s. (**c**) Shows an accelerating frame-rate image series where the time difference between the first two images is 100 μs followed by a difference of 10 μs and then 1 μs. The blue regions in the insets precedes the red ones in time, making the direction of the flow clearly visible. One can also observe that in the last inset the difference is reduced to a pixel or subpixel level, indicating that the atomization process indeed occurs on the ~ 1 μs timescale.

Accurately tracking the evolution of a transient event requires the ability to visualize both the fast- as well as the slow moving parts of the sample. This information can be attained through either long image sequences or a non-linear interframe time. The system’s versatility in the time domain allows for arbitrary frame timestamps, granting parallel access to events that occur in the regimes ranging from $10^0$ to $10^{-7}$ s. This versatility is illustrated with a series of images with a constant inter-frame time (Fig. 7a), a PIV-inspired sequence (Fig. 7b.) and a series with an accelerating frame rate (Fig. 7c).

The red lines of Fig. 7a show the progression of the injection front as a function of time, superimposed on each other as the image series proceeds. The insets in Fig 7b depicts the absolute difference between the two adjacent images where the green shading emphasizes the evolution of the injection. Using this PIV arrangement, the speed on the injection front was approximated to about 40 m/s at both of the indicated times. The insets labeled “difference” in Fig. 7c depict the evolution of the front for two different inter-frame times, where the blue regions temporally precedes the red ones, highlighting the direction of the flow. In the second inset, these regions are reduced to the pixel or sub-pixel level, thus validating the ~ 1 μs time needed to temporally freeze the flow for this injector. Given the images’ spatial resolutions of (12.3lp/mm:312)$_{X,Y}$, and a high shadowgraphy contrast, this approach of varying the temporal properties of the pulse train allows for a thorough quantitative investigation into the flow dynamics of the injection.

## Discussion

In summary, we have presented a FRAME illumination system based on fast pulsed LED technology, allowing for high spatial resolution transmission imaging on the MHz timescale. Each individual LED illuminates a high frequency transmission grating with different orientations, in order to spatially code the individual light pulses. These periodic patterns are then optically relayed onto the sample and the transmitted light recorded in parallel by the sensor. Because all pulses are recorded in one exposure, there is no need for fast readout, allowing the illumination system to be combined with, in principle, any camera. To the best of the author’s knowledge, this is the fastest system capable of MHz imaging without relying on either pulsed lasers or fast detectors.

The optical configuration has several technical advantages over high speed imaging systems based on pulsed lasers or fast detectors. First, since the LEDs are controlled individually, the pulse train can be arranged non-linearly in time and thereby adapt the interframe time to the temporal evolution of the sample. This possibility can compensate for the currently relatively low sequence depth. Second, contrary to pulsed lasers otherwise employed with illumination-based high-speed imaging systems, LED illumination is harmless, less expensive and compact. Third, FRAME naturally provides suppression of background light and multiple light scattering, allowing for shadowgraphy imaging of objects that emit light or are hidden in turbid media. Moreover, the use of incoherent light is beneficial for transmission imaging as speckle patterns and diffraction effects are avoided. Finally, unlike most passive-based high-speed imaging techniques, the high pixel density and field of view is maintained even at the highest frame rates.

Currently the maximum temporal performance of the presented system has been shown to be (876ns:4)$_T$, i.e. 4.56Mfps. Image sequences acquired at even faster rates—5 and 10 MHz—were shown to maintain the spatial performance of either (12.3lp/mm:312)$_{X,Y}$ or (23.9lp/mm:286)$_{X,Y}$. The illumination system will hence directly benefit from any technological advancement towards faster and/or brighter LEDs, opening up for even shorter sequence lengths while still retaining the original FOVs. Brighter LED sources may also allow for longer sequence depths, given that the beam-splitter arrangement is upgraded to facilitate more pulses. Such technological advancement is not unrealistic, as the brightness vs cost of LED technology has been observed to progress in an exponential manner akin to Moore’s law for transistor size—the LED counterpart being dubbed Haitz law^34^.

For the sake of clarity we have chosen to illustrate the capabilities of the system with a fuel port injector, as it combines fast events with fine spatial details. However, as long as the spatial modulations can be imaged effectively, this system can be applied in other transmission imaging situations, such as flow visualization^35^, shockwave imaging^36^ and laser ablation^37^.

The development towards high-speed video cameras that are faster while still providing high spatial resolution is indicative of the growing need to visually track the motion of objects on a sub-microsecond timescale. However, at such (or faster) imaging speeds, few objects have a sufficient spontaneous luminosity needed for visualization, with the exception of high-speed imaging of laser pulses^25,27,38,39^. The presented work demonstrates a new opportunity for videography on the sub-microsecond timescale, uniting slow, high resolution cameras with illumination-aided imaging concepts, allowing for the use of accessible imaging hardware to facilitate investigations of high-speed events.

## Supplementary information

Supplementary information.
