# Supplementary material for: Beyond MHz image recordings using LEDs and the FRAME concept
Source: Sci Rep. 2020 Oct 6;10:16650. doi: 10.1038/s41598-020-73738-1 (PMC7539007; doi:10.1038/s41598-020-73738-1)
Supplement: Supplementary file 1 — Supplementary information. [file 41598_2020_73738_MOESM1_ESM.pdf]

Beyond MHz image recordings using LEDs and the FRAME concept

Vassily Kornienko[1*], Elias Kristensson[1+], Andreas Ehn[1+], Antoine Fourriere[2+], and Edouard Berrocal[1+]

[1]Lund University, Department of Combustion Physics, Lund, 223 63, Sweden
[2]Federal Institute for Geosciences and Natural Resources (BGR), Hanover, Germany
[*]vassily.kornienko@forbrf.lth.se
[+]these authors contributed equally to this work

Supplementary Information

1. Modulation Depth

A Ce:YAG LED acts as the white light source for each channel. Proposed in 1967 but then patented in 1999, a strong blue LED light source excites the cerium atoms in the phosphor which then emit visible light from blue to red (the blue line in Supp. Fig. 1a.). This wide spectrum can cause chromatic abberations upon imaging of the Ronchi gratings, seen as a decrease in modulation depth of the spatial modulation, a fact worth investigating.

In order to look into this, three different spectral filters were placed in the beam path; a longpass filter above 470 nm, a green bandpass filter with a central wavelength of 562nm and bandwidth 40nm, and a very thin one centered around 532 nm called a laserline filter in this section. The quota of the light intensity that is left after applying the filter is given as the q-value in the legend of Supp. Fig. 1a.

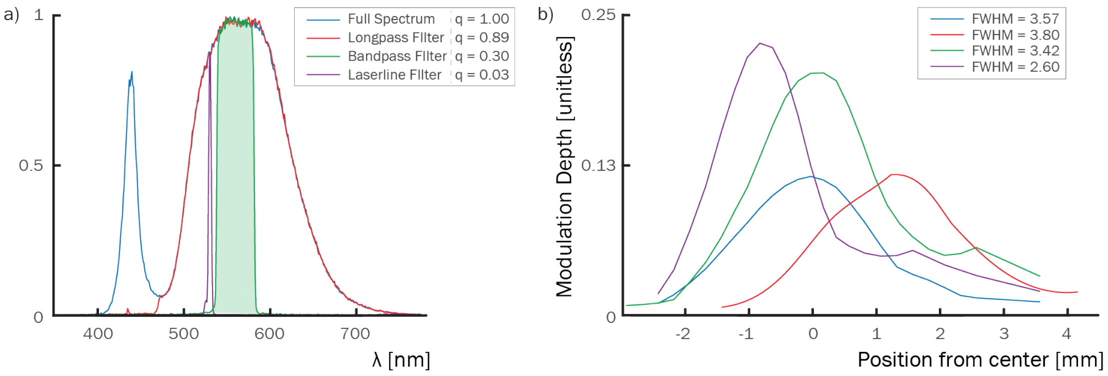

Supp. Fig 1. Spectral filters affect the shape of the image focus. a) Shows the spectrum that is left after placing a given filter in the beam path. The q-value is the amount of light intensity that is left after the filter, where the light green shading represents the amount of light that is left after the bandpass filter. b) shows that the spectral filters increase the modulation depth of the image of the Ronchi grating and reduce the depth of focus as they constrain the bandwidth of light that is imaged by the system.

Images of a 2D spatial modulation were taken at various positions along the optical axis (z-position). The modulation depth was then calculated by taking the maximum value of the modulation's corresponding cluster divided by the maximum value of the DC component in the Fourier domain. Due to the circular symmetry of the optical system, the information gathered from a spatial modulation in one direction can be generalized for the whole fourier domain. A conversion to actual modulation depth (half the amplitude of the corresponding sine function divided by its mean) was then performed. As one could expect the thinnest filter gave the largest modulation depth and shortest depth of focus (Supp. Fig. 1b). However the light intensity decreased by a factor of 50, creating problems with low signal to noise ratios. The bandpass filter gave a similar modulation depth without as much loss of light, followed by the longpass filter. These investigations point to a trade off between the amount of light (higher signal to background ratio in the context of shadowgraphy) and the portion of information that is shifted to the individual modulations as compared to the DC component. Even though only one third of the light remains after the bandpass

filter, while the increase in modulation depth is only about two times that of no filter, the bandpass filter was used for most measurements.

In conclusion, the incorporation of spectral filters has no obvious advantage over no filters. The choice depends on how much light intensity one needs for a certain type of measurement and if the slight increase in optical sectioning and spatial resolution is worth the decrease in signal.

2. Spatial Resolution

The finite information storage capacity of a camera causes a trade-off between the number of frames and the spatial resolution that is attainable using FRAME. Another way of visualizing this effect is that in order to isolate single frames, a low pass filter needs to be applied in the Fourier domain, decreasing the effective number of pixels used for a given image. In order to evaluate the spatial resolution of the modulated images, a Sector Star target (Thorlabs Sector Star 10) was imaged (Supp. Fig. 2a) and extracted (Supp. Fig. 2b) under identical conditions to the two different fields of view used in these experiments. The cutoff frequency of the individual images, defined as the frequency that is only transferred at a contrast level of 15.3% (the Rayleigh criteria), was then calculated and identified as the attainable spatial resolution of the given image.

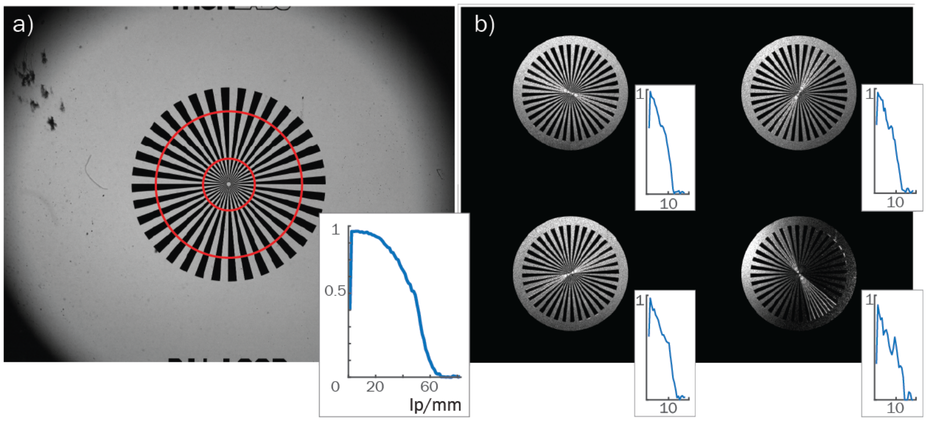

Supp. Fig 2. Spatial resolution before and after extraction. a) shows the raw image and its corresponding modulation transfer as a function of modulation frequency. The red lines depict two example radii at which the MTF is extracted. A specific component of the fourier transform of the 1D square function along the profile of the red circles, gives the MTF at that star spoke frequency. b) is the series of extracted images with their corresponding MTF curves. The values are much lower than for the original image due to the low pass filtering in the Fourier domain.

Note that the star target is quite a difficult object to image using FRAME. This since the spatial modulations will spatially beat with the spokes of the star that are adjacent in angle (compare to heterodyne detection, except in spatial coordinates instead of time). This can be seen as some low frequency modulations and lighter parts that arise in the final images of Supp. Fig. 2b. High spatial resolution in these directions is not possible to image using FRAME, however most objects do not have as difficult a structure as a Sector Star target, it is in fact not a natural image. In the case of the injector nozzle, the beating artifacts visible in the Sector Star figure would arise if we were to use a near horizontal spatial modulation for one of the light pulses. This is equivalent to the argument made in the main text that we should place the clusters away from any recurring static structure in the fourier domain.

The following table summarizes the values that were extracted for the large and small FOVs. We have decided to also extract the MTF with and without the spectral filter to see how this factor affects the final spatial resolution. Furthermore the last row of the tables are the cutoff frequencies when the spectrally filtered image is background corrected and histogram adjusted with a threshold of 3%.

Table 1. The spatial resolution considerations for the small FOV. Values are given in lp/mm.

	**Raw Image**	**Image 1**	**Image 2**	**Image 3**	**Image 4**	**Mean**
**Without Filter**	*59.9*	22.5	22.4	22.5	22.5	22.48
**With Filter**	*60.6*	24.4	24.4	23.3	23.4	23.90
**Background Corr.**		24.8	24.6	24.1	23.4	24.20

Table 2. The spatial resolution considerations for the large FOV. Values are given in lp/mm.

	Raw Image	Image 1	Image 2	Image 3	Image 4	Mean
**Without Filter**	*57.6*	12.5	12.8	11.7	12.7	12.43
**With Filter**	*58.6*	12.5	13.0	12.4	11.5	12.35
**Background Corr.**		12.8	13.2	12.1	13.5	12.90

There are a few things to note when it comes to how the spatial resolution varies depending on the measurement:

1. **Application of Spectral Filter:** Except for image four on the second row of Table 2 there is a general trend that upon the application of the spectral filter the spatial resolution increases slightly. This comes from the fact that with a higher modulation depth more of the light goes into the fourier clusters allowing for higher contrast for all frequencies. This would entail that higher spatial frequencies are visible above the noise floor when the spectral filter is applied.

2. **Background Correction:** When performing a background correction there is again a general trend of a slightly higher spatial resolution. A background correction followed by a histogram correction increases the contrast of an image. Hence by the same reasoning as point 1, this would entail a slight increase in spatial resolution.

3. **Raw Image Resolution:** For the large FOV the raw image has a higher spatial resolution than the four extracted images combined. This is expected since when filtering the clusters in the fourier domain with circularly symmetric filters there will be space in between, i.e. all the available spatial resolution has not been used. However for the small FOV the raw image has a considerably smaller value than the four individual images combined. The reasoning for this is that the optical setup has a limiting resolution that is lower than the camera's, hence the four images combined will result in a higher spatial resolution than if a single image is captured with the setup.

3. Preservation of spatial resolution with speed

Since passive techniques rely on fast readout speed of photoactive elements, they usually suffer from a loss of spatial resolution as a function of speed. Moving the speed characteristics to the illumination side, as is done with FRAME, releases this constraint, allowing for higher speeds to benefit from the same spatial resolution as their slower counterparts. Specifically to the (non-temporally resolved) 5 and 10 MHz videos, the spatial resolution does not differ as compared to the table in the above section. Supp. Fig. 3 shows just this, that the spatial resolution stays close to flat with pulse length, directly correlating to videography speed.

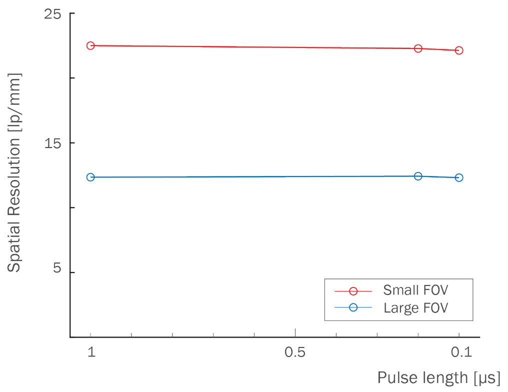

Supp. Fig 3. Preservation of spatial resolution with increasing videography speed. The spatial resolution as a function of pulse length is flat for both the small and large FOV. The small dip comes from a loss of pulse train stability, covering the highest spatial frequencies in shot noise.

4. Temporally Resolved Pulse Trains for Videography and Pulse Train characteristics

In passive videography with high speed digital cameras, the maximum frame-rate is naturally the inverse of the frame exposure time, with some deviation due to readout or shifting times. After all, the electrons that correspond to a given frame must be moved out of the way before the next image is acquired. This is not necessarily the case with illumination based imaging techniques, where the individual pulses of a pulse train can, and most often will, overlap in time to a certain extent. Therefore

one can see higher imaging rates than the individual pulse lengths. In the literature there have been attempts but no general consensus on what constraints to apply to these pulse trains in order to call them temporally resolved. Therefore, for these measurements, we have tried to define temporal resolution from the bottom up. The purpose of videography is to follow the temporal evolution of an event with images. In more general terms, videography is a method of sampling the time-axis, where each discrete sample corresponds to an image. The temporal width over which the event is integrated within a single sample is what sets the limit to the sampling rate (compare to sampling spatial properties with the pixels of a camera). Indeed if one were to sample the time-axis with delta functions then a continuous discretization of the temporal evolution of the event would be possible, corresponding to an infinite sampling rate/videography speed. With this in mind, it is clear that the sampling rate of a videography system is set by the temporal gate function of the acquired image frame.

In the context of active videography, the temporal characteristics of a single illuminating pulse is what sets the discretization step-size of the time-axis - more specifically the pulse length corresponds to the minimum discretization step. In order to determine a suitable pulse length for an arbitrary pulse we turn to Gaussian pulse profiles. It can be shown that 76% of a Gaussian pulse's total power resides within the limits of its FWHM (inset of Supp. Fig. 4a). This value of 76% power is the value we will use to define an illuminating pulse's pulse length, or equivalently our discretization step size. Placing multiple gaussians such that their half maxima coincide discretizes the time-axis with slots that are $\Delta =$FWHM in size (Fig. 4a). This definition of pulse length Δ, within which 76% of the pulse power is concentrated, limits oversampling of the time axis, assures a 76% degree of uniqueness to every image and can be generalized to any frame profile. For the system presented herein, the temporal profile of the frames are dependent on the LED pulse shape, where an example is shown in the inset of Supp. Fig. 4b (a longer pulse than that shown in the main text). Here an upper and lower limit have been extracted within which 76% of the pulse power lies. In an equivalent way as with the Gaussians, placing them such that these limits coincide sets the maximum sampling rate of the time-axis.

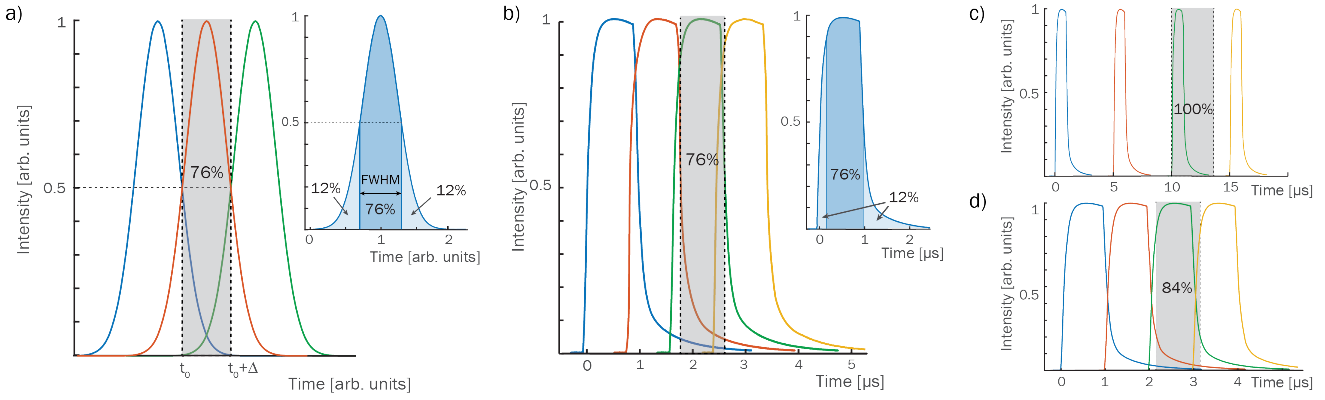

Supp. Fig 4. Temporally resolved pulse trains. Defining the maximum temporal resolution of a pulse train entails defining a minimum attainable discretizing step size of the time-axis. To do this we look into Gaussian profiles. 76% of a Gaussian's power lies within its FWHM (inset of a). Hence placing these in a pulse train such that their half maxima limits coincide (a), assures 76% temporal uniqueness to the individual images. For example, the time-slot $[t, t+\Delta]$ is dominated by the red pulse, hence the image information arising from the red pulse will be temporally unique to all others. We use this Δ as the discretization step-size. (b) shows an example pulse generated by the system presented herein where limits have been indicated to show where 76% of the pulse's power resides. Creating a pulse train in an identical manner to the Gaussians of (a), i.e. sampling the time-axis with a step size bounded by the 76% limit, results in a 1.22MHz video of four images. (c) is a 200kHz pulse train where we obviously see that the pulses are temporally resolved, indeed they do not overlap at all, leaving a 100% temporal uniqueness to each image. (d) is instead a 1MHz pulse train. Here we see that sampling the time-axis in this way, leaves us with 84% temporal uniqueness per image.

This value of 76% temporal uniqueness is a defined minimum limit for the discretization of the time-axis with a certain pulse shape. Indeed anything above it will also be temporally resolved according to our definition. For example the pulse train used to sample the time-axis at a rate of 200kHz (Fig. 4c) allows for 100% temporal uniqueness to each time-slot, which is obviously above the resolution limit. If one were to take an image series at 1MHz using the same pulses (Fig. 4d), a temporal uniqueness of 84% per time-slot will be maintained, i.e. it will be able to produce a temporally resolved image series.

5. 10MHz videography and pulse train stability

The LEDs of this illumination unit are intrinsically limited by the lifetime of the Ce:YAG phosphor of about 45ns. However the limiting factor for this specific system is the speed of the trigger electronics. Going below a pulse length of 200ns the success rate of the LED pulses decreases rapidly. At 100ns pulse lengths the individual LEDs only pulse with an adequate amount of light about 50% of the time, hence for a pulse train of four pulses it has a stability of 12.5%. Furthermore, the pulses are not temporally resolved under the aforementioned criteria as we can only reach a temporal uniqueness of 41.2%. Supp. Fig. 5 show the difference between a successful (a) and unsuccessful (b) event. The success of the video can be directly seen as an obvious decrease in SNR in the corresponding unsuccessful frame, seen as the top image of the inset of Supp. Fig. 5b.

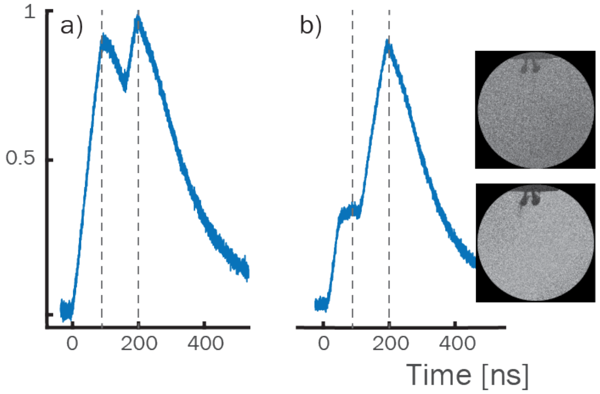

Supp. Fig 5. Stability of 100ns pulse trains. a) shows the signal for a successful 10MHz two pulse train. The temporal contrast is at 11.7%, hence not quite temporally resolved according to our definitions. b) is an example of an unsuccessful event. Here it is obvious that we cannot get temporally resolved imaging. This effect is also visible in the SNR of the final images, where the upper one corresponds to the unsuccessful pulse

This fact goes to show that the setup will directly benefit from an advancement in LED technology, a field that has and will keep improving very fast[34].

6. Stray Light Suppression

It can be difficult to image an event in shadowgraphy mode if the sample emits a lot of light, since the dark shadows will unavoidably light up. By tagging the light pulses with spatial modulations, only the emitted light within the pulse time will be shifted in the Fourier domain. Hence, stray light suppression is a natural consequence when imaging with FRAME. This is the reason as to why the camera exposure can be made long compared to the pulse train length.

In order to illustrate this, the ignition of a regular match was imaged with the setup (Supp. Fig. 6). The total camera integration time was set to 7ms and the modulated light pulses had a pulse length of 20μs, contributing to a total of 20% of the total light incident on the camera during a single exposure. The panel b) is the image extracted from the center cluster in the fourier domain, the DC component. Equal SBRs between the raw image (Supp. Fig. 6a.) and the image stored within the DC component (Supp. Fig. 6b.) prove that the stray light is stored therein. Upon extraction of a modulated image (Supp. Fig. 6c.), this extra stray light that came with the ignition process could be suppressed by a factor of ~ 5 (corresponding to the 20% mentioned earlier), essentially allowing the system to "see through" the light emitted from the event.

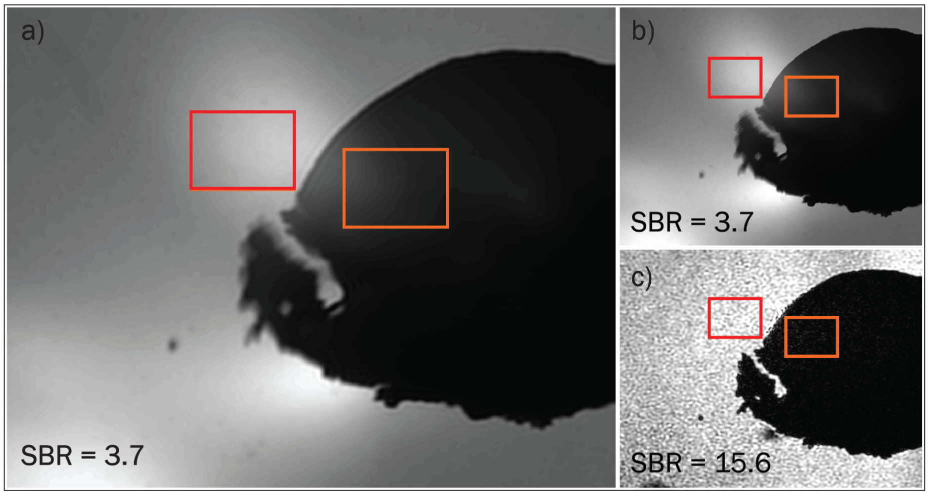

Supp. Fig 6. Illustration of stray light suppression and an increased SBR. a) is the raw modulated image taken of the ignition event. One can see that there is some movement in the extruding tail and stray light is visible even in the parts where the shadow should cause the image to be completely black. The SBR is calculated by taking an average of the values within the red box divided by the average within the orange box. b) is the extracted DC component of the image a), depicting an equal SBR while, allowing the conclusion that the stray light is stored within the DC component. c) is an extracted timestamped image. The SBR has increased by a factor of about 5.

7: LED characteristics

The LED rise time (10% – 90% of full power), was measured to be 205ns (Supp. Fig. 7a). Note that this is an example of a pulse that reaches full power, hence it differs slightly from some of the pulses that do not reach saturation (compare the pulse trains of Supp. Fig. 4b with Supp. Fig. 8). The temporal jitter characteristics of the pulsed LEDs was calculated by measuring the position of the LED pulse in time wrt a global trigger. As for all the experiments presented herein, the LaVision PTU-X, with a temporal jitter of 0.05ns, was used as the trigger source. The LED pulse was measured with a photodiode connected to a Lecroy 6GHz oscilloscope which itself has a temporal jitter of 2.5ps. The time between the trigger pulse and detection of the LED pulse (set to 200ns) was then measured with the Lecroy "skew at level" function. The skew of 25000 such trigger events were histogrammed and the mean was set to zero (Supp. Fig 7b). A temporal jitter FWHM of 34.5ns was then extracted.

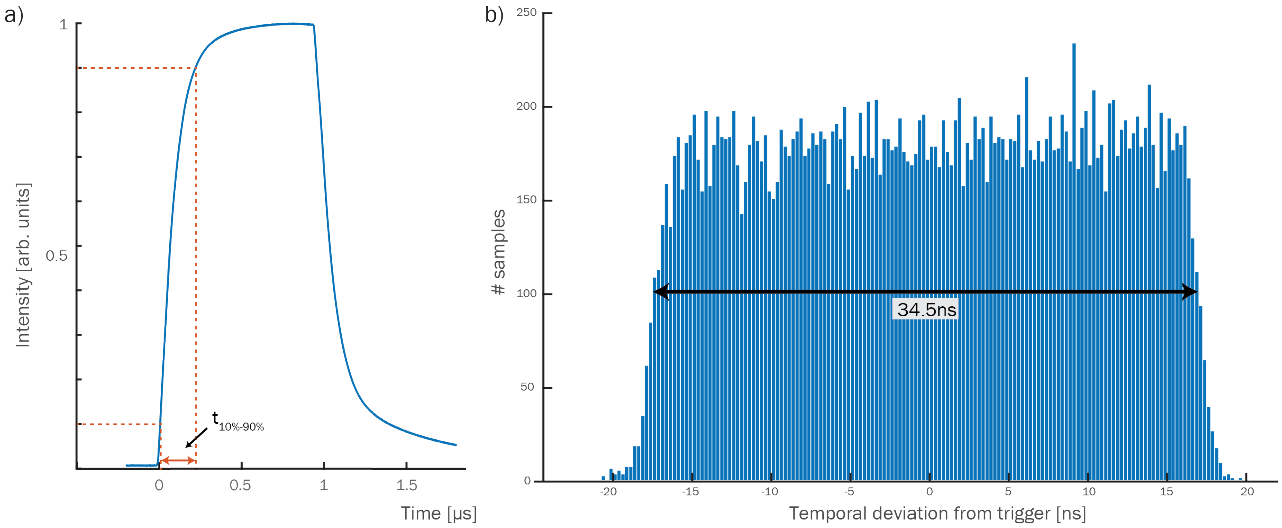

Supp. Fig 7. Temporal characteristics of the pulsed LEDs. a) An individual 1μs pulse resulting in a $10\% - 90\%$ of full power rise time of 205ns for the individual LEDs. b) A histogram of 25000 trigger events measuring the temporal jitter of the pulsed LEDs wrt a global trigger. The histogram has been shifted to a mean of zero. A temporal jitter FWHM of about 34.5ns is extracted.

8: Dynamic Range per Pulse

Since FRAME stores a series of images on a chip within a single exposure, the dynamic range of each pixel will be divided between these individual images (or equivalently their corresponding time-slot). This division can be seen in the raw images where the contrast of the object under investigation varies depending on where on the sensor the shadow lies. Naively, this would mean that each time-slot is allowed a dynamic range of DR/N, where DR is the dynamic range of each pixel, and N is the total number of images stored within the single exposure. In this case $DR = 2^{10} = 1024$, and $N = 4$ leaving $DR/N = 256$ grayscale levels per time-slot. However the allowed overlap between the pulses of the pulse train (discussed in the main text and Supplementary Information 4) will result in a decrease in the dynamic range reserved for a unique time-slot, as compared to 256. Taking this into account, the effective dynamic range, $DR_{\text{eff,t}}$, for a given time-slot t, can be given by:

$$DR_{\text{eff,t}} = \frac{D_{\text{bit}}}{N} \frac{I_t}{I_{\text{tot}}}. \tag{1}$$

Here D_{bit} is the bit depth of the pixels on the sensor, N is the number of temporally unique time-slots within the pulse train and I_{tot} is the total integrated intensity within the time-slot. For example, if the image series is a 4.56MHz series, as shown in Supp. Fig. 8, then the limits of integration are set as the limits of the time-slot (the dashed lines). I_{tot} is hence $I_{t-2} + I_{t-1} + I_t + I + t + 1$, and I_t is the integrated intensity of the frame of interest within its time slot. It can be seen that I_{tot} will be largest for the third time-slot as intensity from all other pulses interfere with it. The dynamic range from this time-slot is the one stated in this text. In conclusion, this equation essentially means that the dynamic range that is reserved to the temporally unique information within a given time-slot decreases by a factor given by the interference from other pulses.

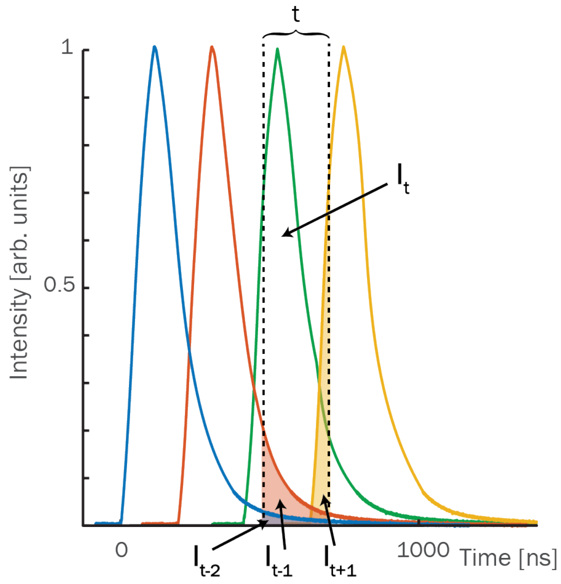

Supp. Fig 8. Calculation of the effective dynamic range of time-slot *t*. The effective dynamic range of time-slot *t* depends on how much light from the other pulses "leaks" into the corresponding pulse's temporally unique window. In the case of the 4.56MHz measurement, the dynamic range decreases by a factor of $I_t/(I_{t-2}+I_{t-1}+I_{t+1}+I_t)$, from 256 to 189 grayscale levels.

For the case of the 1MHz measurement, with the pulse train of Supp. Fig. 4d, an effective dynamic range per time-slot of 214 grayscale levels was determined while an effective dynamic range of 194 grayscale levels was calculated for the 4.56MHz pulse train (Main text Fig. 4c and Supp. Fig. 8). Note that this plays a major role as the sequence depth increases, as one cannot have less than 1 bit dynamic range per image if one wants to extract anything at all.
